# Domestic

**Published:** 1841-10-16

**Authors:** 


### DOMESTIC.

### HEALTH OF THE CITY.

INTERMENTS *in the City and Liberties of Philadelphia, from the 2d of October to the 9th.*

| Diseases. | Adults. | Children. |
|---|---|---|
| Abscess of Liver, | 1 | 0 |
| Apoplexy, | 3 | 0 |
| Bowel Complaint, | 0 | 1 |
| Casualties, | 0 | 2 |
| Croup, | 0 | 1 |
| Chlorosis, | 1 | 0 |
| Colic, | 1 | 0 |
| Consumption of the lungs, | 10 | 3 |
| Convulsions, | 0 | 2 |
| Diarrhœa, | 2 | 1 |
| Dropsy, | 2 | 0 |
| —— abdominal, | 1 | 1 |
| —— Head, | 1 | 1 |
| Disease of Spinal Marrow, | 1 | 0 |
| —— Throat, | 1 | 0 |
| Dysentery, | 2 | 3 |
| Debility | 0 | 4 |
| Enlargement of heart, | 0 | 1 |
| Fever, | 1 | 1 |
| —— Congestive, | 0 | 1 |
| —— Remittent, | 2 | 1 |
| —— Typhus, | 2 | 0 |
| —— Scarlet, | 0 | 2 |
| Gangrene of Lungs, | 1 | 0 |
| Inflammation of the Brain, | 0 | 1 |
| —— Bronchi, | 1 | 1 |
| —— Lungs, | 2 | 0 |
| —— Stomach and Bowels, | 0 | 1 |
| *Carried forward,* | 35 | 28 |

| Diseases. | Adults. | Children. |
|---|---|---|
| *Brought forward,* | 35 | 28 |
| Inflammation of Bowels, | 1 | 1 |
| Intemperance, | 1 | 0 |
| Inanition, | 0 | 1 |
| Marasmus, | 1 | 5 |
| Old age, | 1 | 0 |
| Palsy, | 2 | 0 |
| Scrofula, | 0 | 1 |
| Small pox, | 2 | 3 |
| Still-born, | 0 | 8 |
| Tabes Mesenterica, | 0 | 1 |
| Trismus, | 0 | 1 |
| Ulcerated Sore Throat, | 0 | 1 |
| Unknown, | 1 | 0 |
| Total, 94— | 44 | 50 |

Of the above, there

| | |
|---|---|
| were under 1 year, | 22 |
| From 1 to 2 | 6 |
| 2 to 5 | 15 |
| 5 to 10 | 4 |
| 10 to 15 | 2 |
| 15 to 20 | 1 |
| 20 to 30 | 11 |
| 30 to 40 | 9 |
| 40 to 50 | 7 |
| 50 to 60 | 7 |
| 60 to 70 | 3 |
| 70 to 80 | 5 |
| 80 to 90 | 2 |
| Total, | 94 |

Of the above there were 5 from the alms-house, 10 people of colour, and 1 from the country, which are included in the total amount.

